# Supplementary material for: Early life stress induces social behavioral deficits and peripheral biomarker alterations in adolescence that perpetuate intergenerationally
Source: bioRxiv. 2025 Dec 1:2025.11.27.690841. Preprint. [Version 1] doi: 10.1101/2025.11.27.690841 (PMC12694602; doi:10.1101/2025.11.27.690841)
Supplement: Supplement 2 [file media-2.pdf]

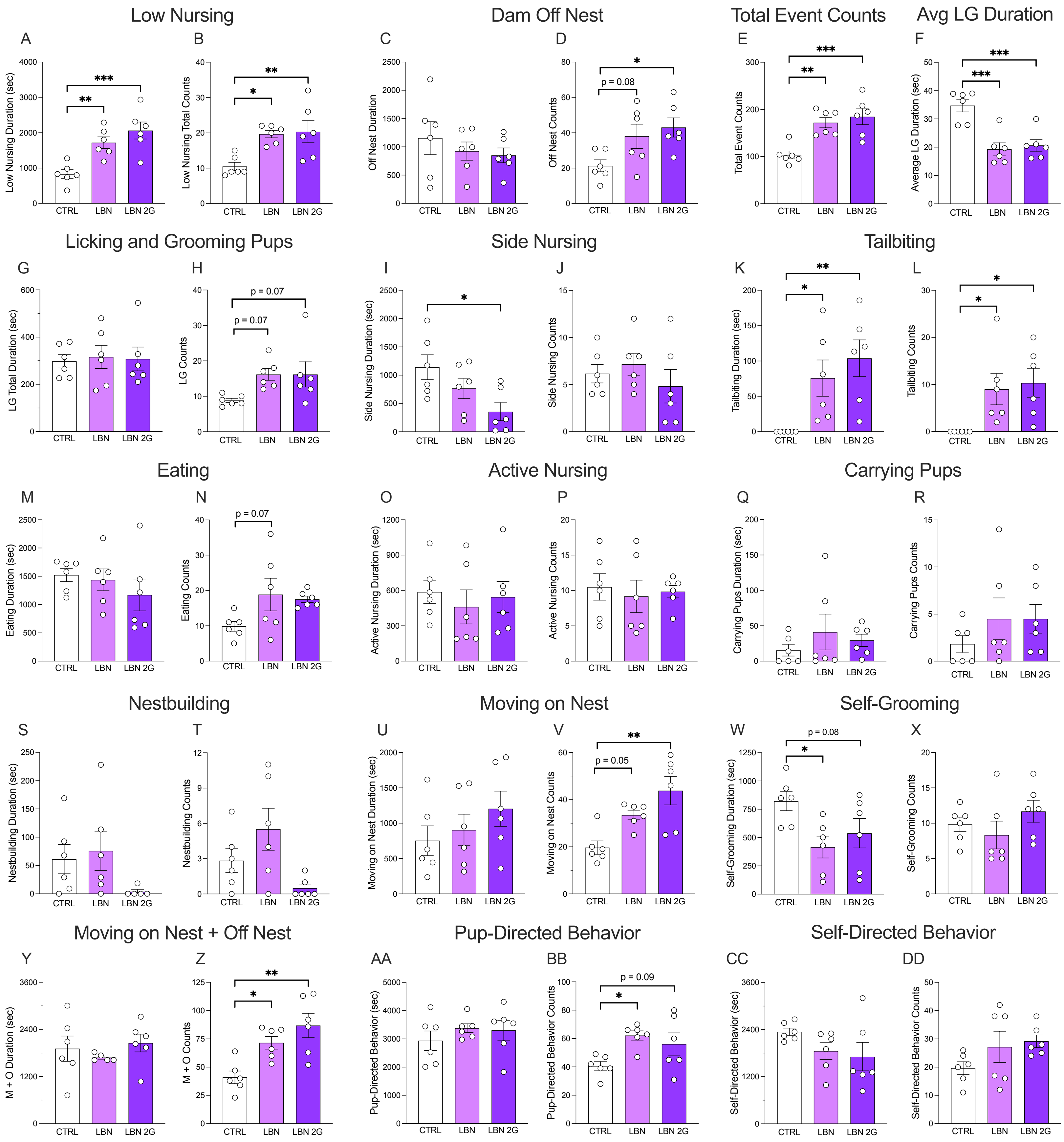

Low Nursing
A
Low Nursing Duration (sec)
**
***
B
Low Nursing Total Counts
*
**
Dam Off Nest
C
Off Nest Duration
D
Off Nest Counts
p = 0.08
*
Total Event Counts
E
Total Event Counts
**
***
Avg LG Duration
F
Average LG Duration (sec)
***
***
Licking and Grooming Pups
G
LG Total Duration (sec)
H
LG Counts
p = 0.07
p = 0.07
Side Nursing
I
Side Nursing Duration (sec)
*
J
Side Nursing Counts
Tailbiting
K
Tailbiting Duration (sec)
*
**
L
Tailbiting Counts
*
*
Eating
M
Eating Duration (sec)
N
Eating Counts
p = 0.07
Active Nursing
O
Active Nursing Duration (sec)
P
Active Nursing Counts
Carrying Pups
Q
Carrying Pups Duration (sec)
R
Carrying Pups Counts
Nestbuilding
S
Nestbuilding Duration (sec)
T
Nestbuilding Counts
Moving on Nest
U
Moving on Nest Duration (sec)
V
Moving on Nest Counts
p = 0.05
**
Self-Grooming
W
Self-Grooming Duration (sec)
*
p = 0.08
X
Self-Grooming Counts
Moving on Nest + Off Nest
Y
M + O Duration (sec)
Z
M + O Counts
*
**
Pup-Directed Behavior
AA
Pup-Directed Behavior (sec)
BB
Pup-Directed Behavior Counts
*
p = 0.09
Self-Directed Behavior
CC
Self-Directed Behavior (sec)
DD
Self-Directed Behavior Counts
CTRL
LBN
LBN 2G
