# Supplementary material for: Early life stress induces social behavioral deficits and peripheral biomarker alterations in adolescence that perpetuate intergenerationally
Source: bioRxiv. 2025 Dec 1:2025.11.27.690841. Preprint. [Version 1] doi: 10.1101/2025.11.27.690841 (PMC12694602; doi:10.1101/2025.11.27.690841)
Supplement: Supplement 3 [file media-3.pdf]

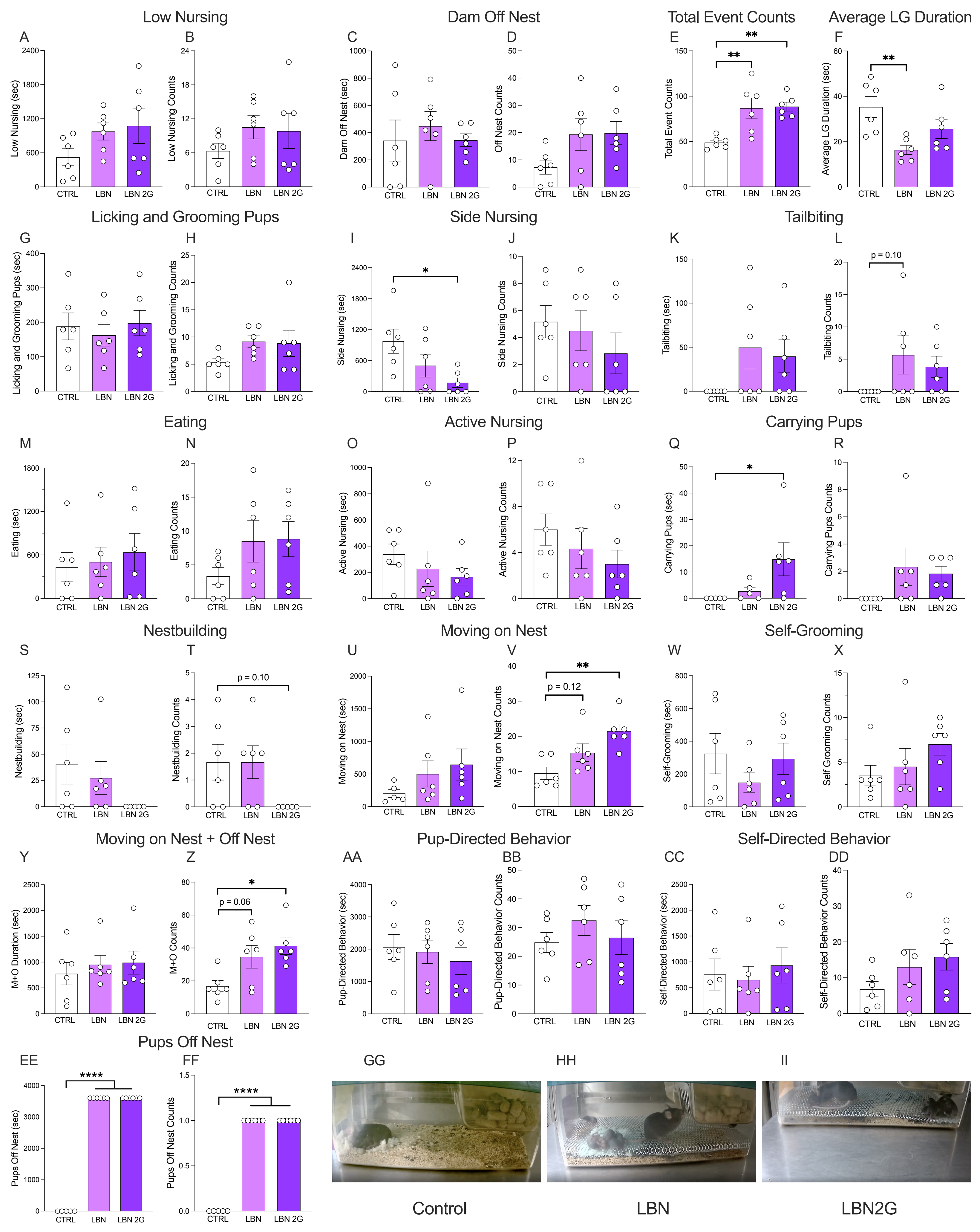

Low Nursing
A
Low Nursing (sec)
CTRL
LBN
LBN 2G
B
Low Nursing Counts
Dam Off Nest
C
Dam Off Nest (sec)
D
Off Nest Counts
Total Event Counts
E
Total Event Counts
**
**
Average LG Duration
F
Average LG Duration (sec)
**
Licking and Grooming Pups
G
Licking and Grooming Pups (sec)
H
Licking and Grooming Counts
Side Nursing
I
Side Nursing (sec)
*
J
Side Nursing Counts
Tailbiting
K
Tailbiting (sec)
L
Tailbiting Counts
p = 0.10
Eating
M
Eating (sec)
N
Eating Counts
Active Nursing
O
Active Nursing (sec)
P
Active Nursing Counts
Carrying Pups
Q
Carrying Pups (sec)
*
R
Carrying Pups Counts
Nestbuilding
S
Nestbuilding (sec)
T
Nestbuilding Counts
p = 0.10
Moving on Nest
U
Moving on Nest (sec)
V
Moving on Nest Counts
**
p = 0.12
Self-Grooming
W
Self-Grooming (sec)
X
Self Grooming Counts
Moving on Nest + Off Nest
Y
M+O Duration (sec)
Z
M+O Counts
*
p = 0.06
Pup-Directed Behavior
AA
Pup-Directed Behavior (sec)
BB
Pup-Directed Behavior Counts
Self-Directed Behavior
CC
Self-Directed Behavior (sec)
DD
Self-Directed Behavior Counts
Pups Off Nest
EE
Pups Off Nest (sec)
****
FF
Pups Off Nest Counts
****
GG
HH
II
Control
LBN
LBN2G
