# Supplementary figures and images for: Early life stress induces social behavioral deficits and peripheral biomarker alterations in adolescence that perpetuate intergenerationally
Source: bioRxiv. 2025 Dec 1:2025.11.27.690841. Preprint. [Version 1] doi: 10.1101/2025.11.27.690841 (PMC12694602; doi:10.1101/2025.11.27.690841)

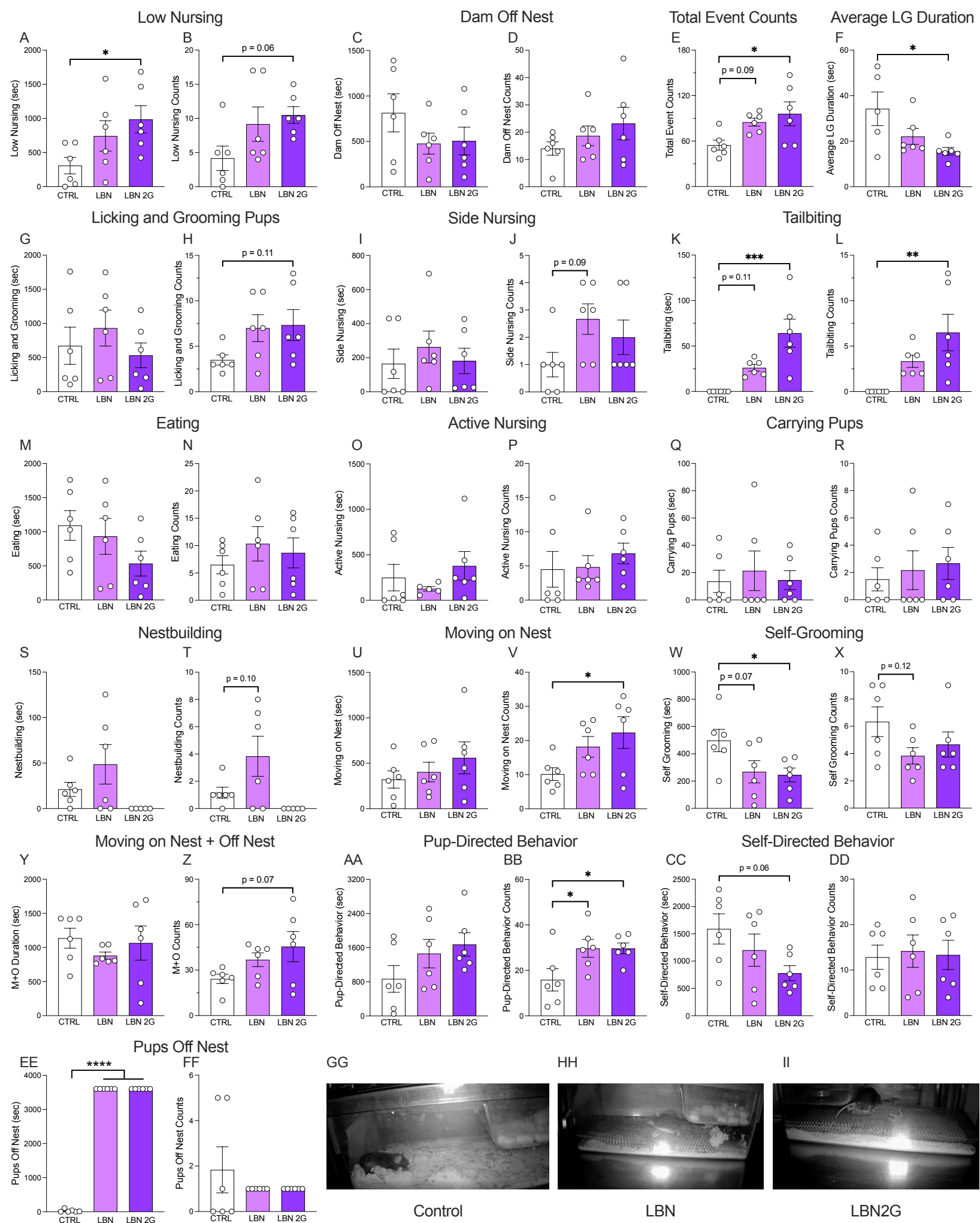

Supplement: Supplement 4 [file media-4.pdf]

Pups per litter

15  
10  
5  
0

CTRL

LBN

LBN  
2G

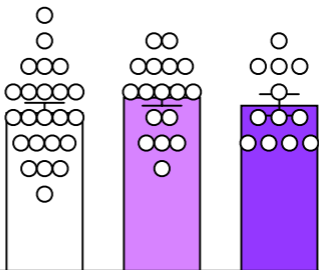

Supplement: Supplement 5 [file media-5.pdf]

**A**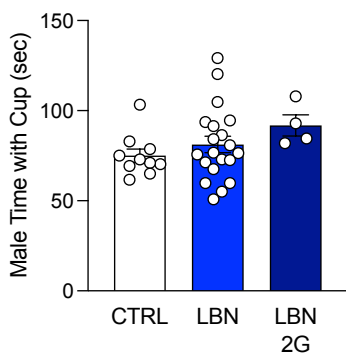**B**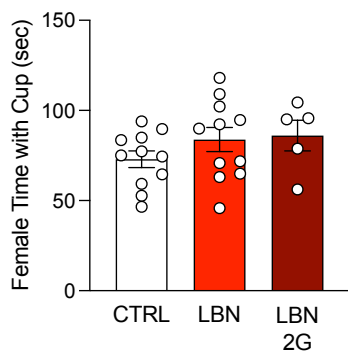**C**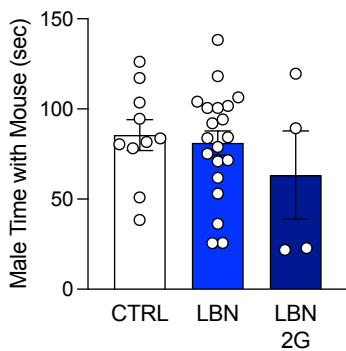**D**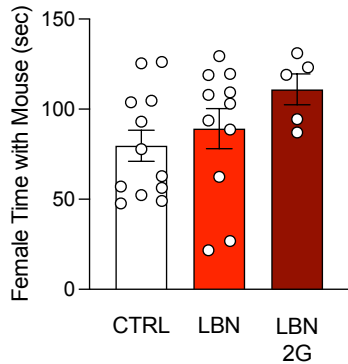**E**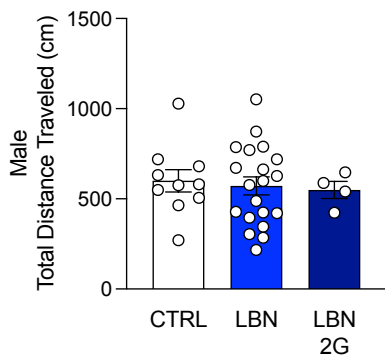**F**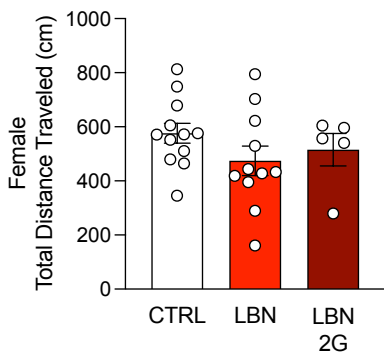**G**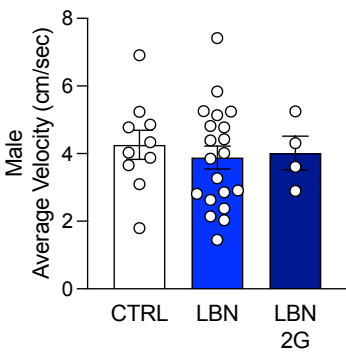**H**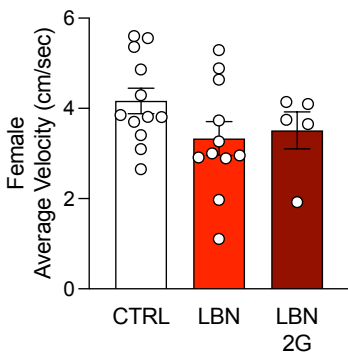

Supplement: Supplement 7 [file media-7.pdf]

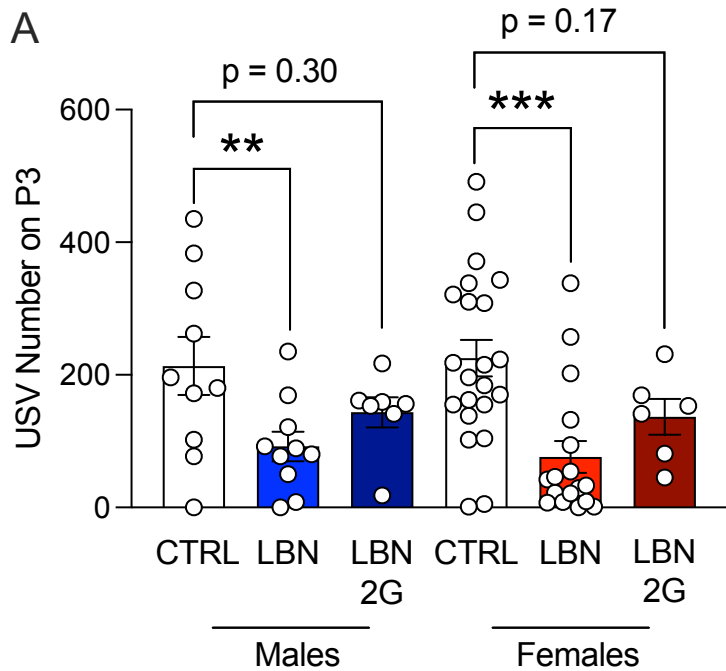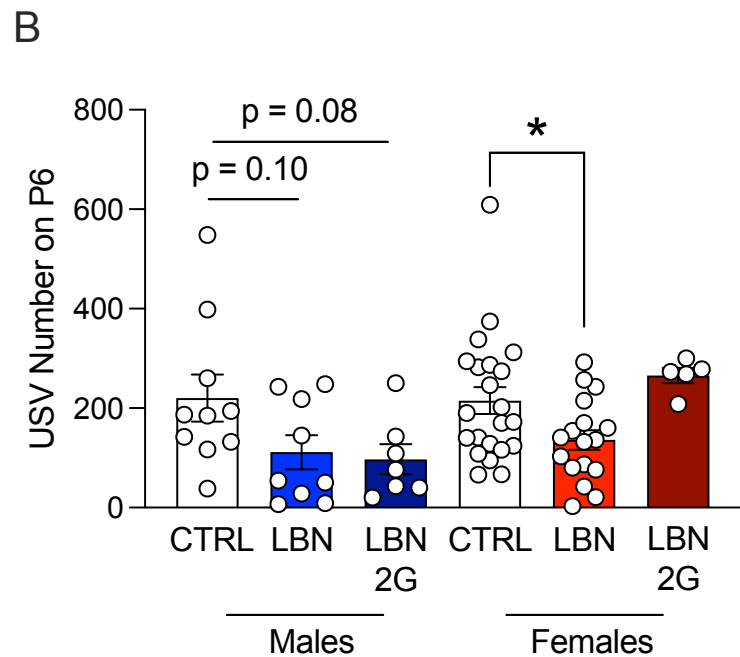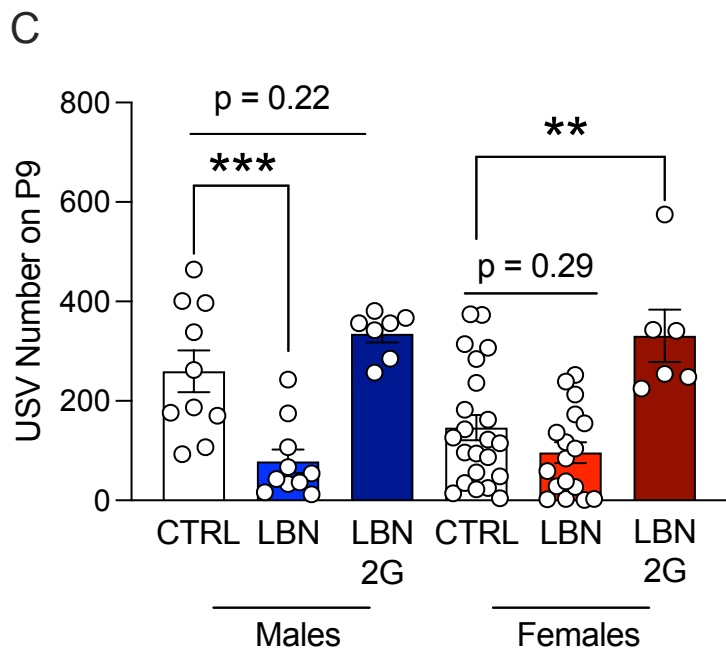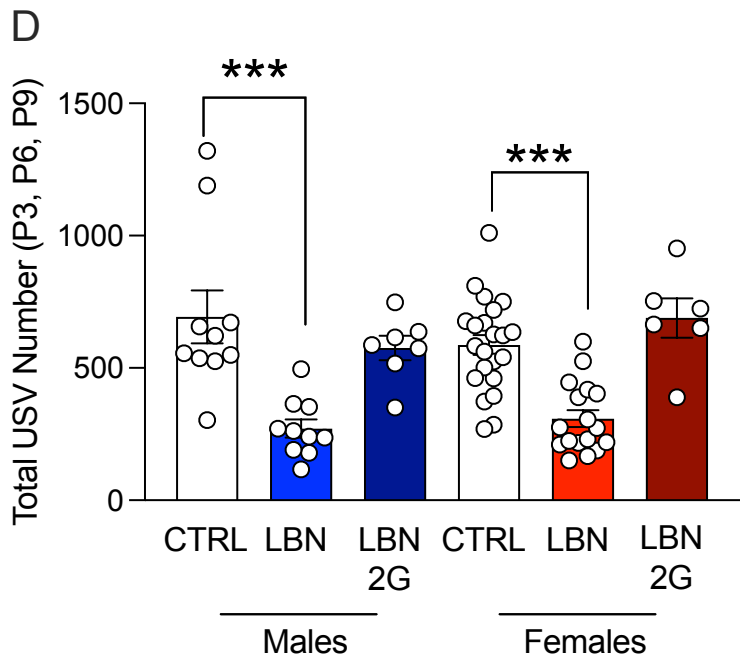

Supplement: Supplement 8 [file media-8.pdf]

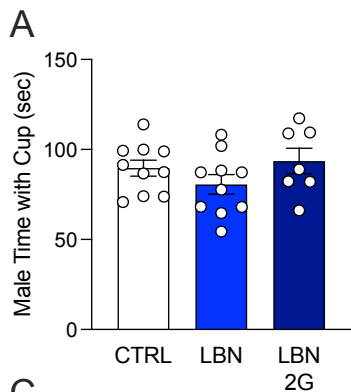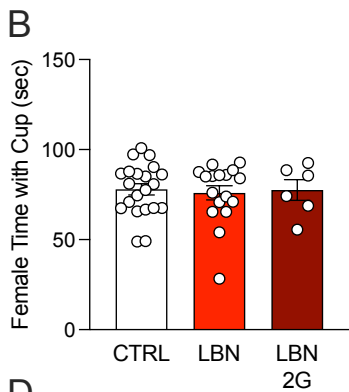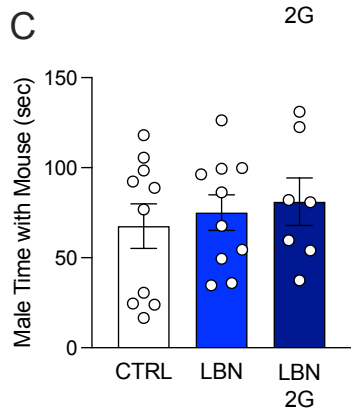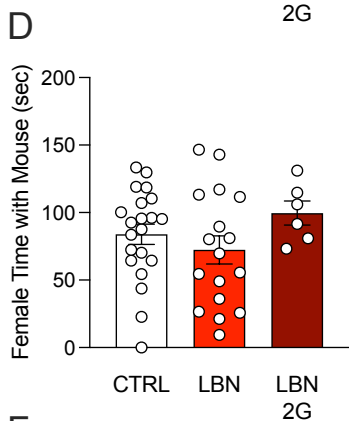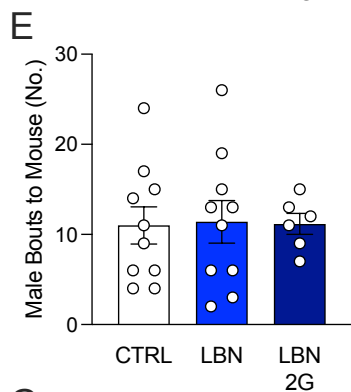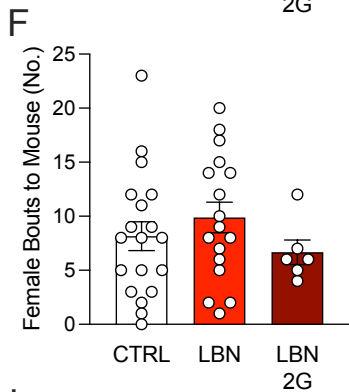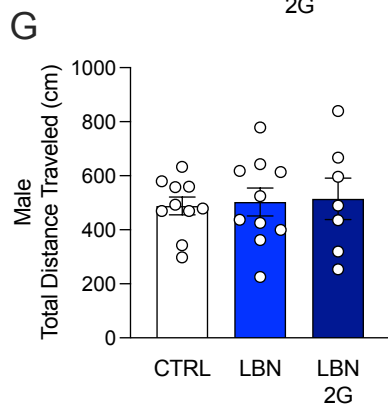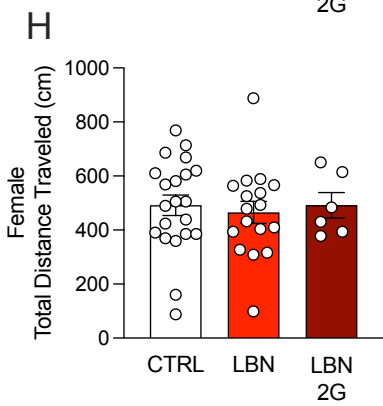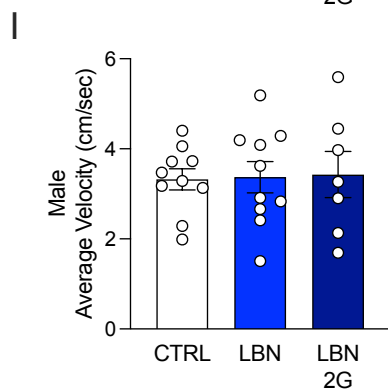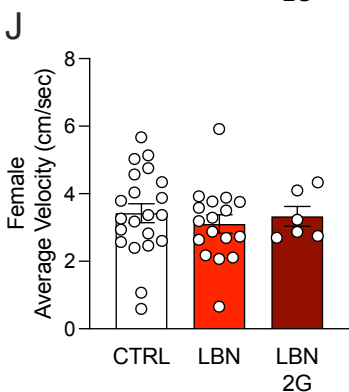

Supplement: Supplement 9 [file media-9.pdf]

A

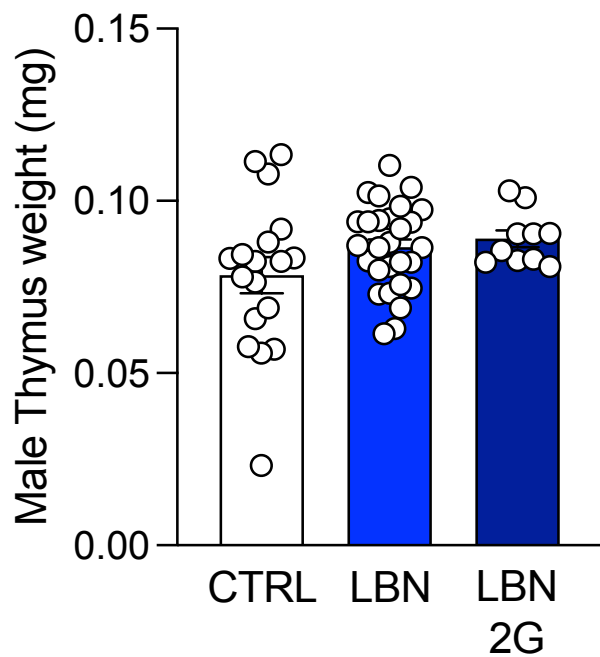

B

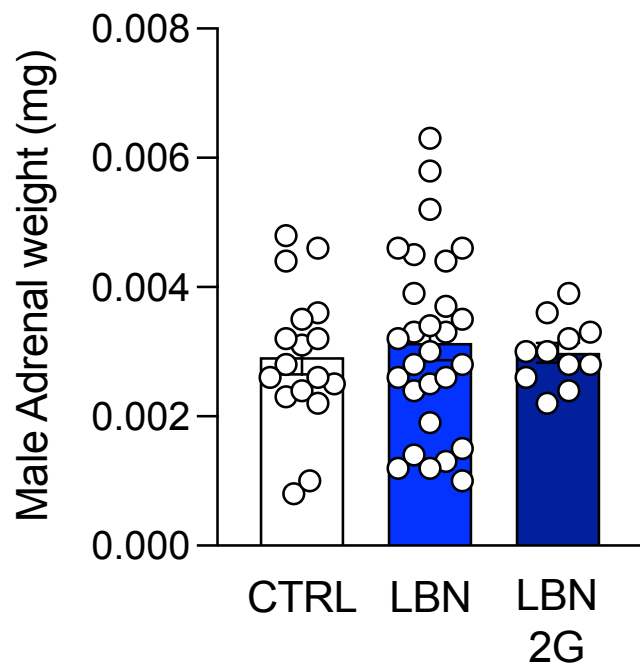

C

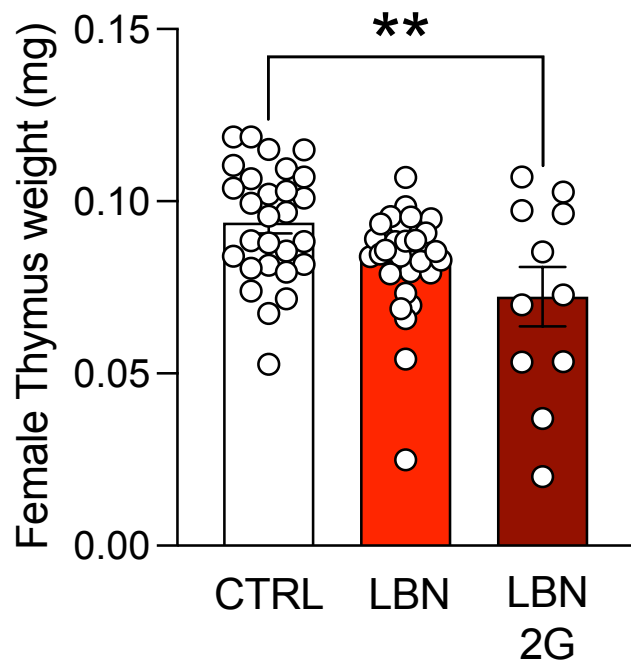

D

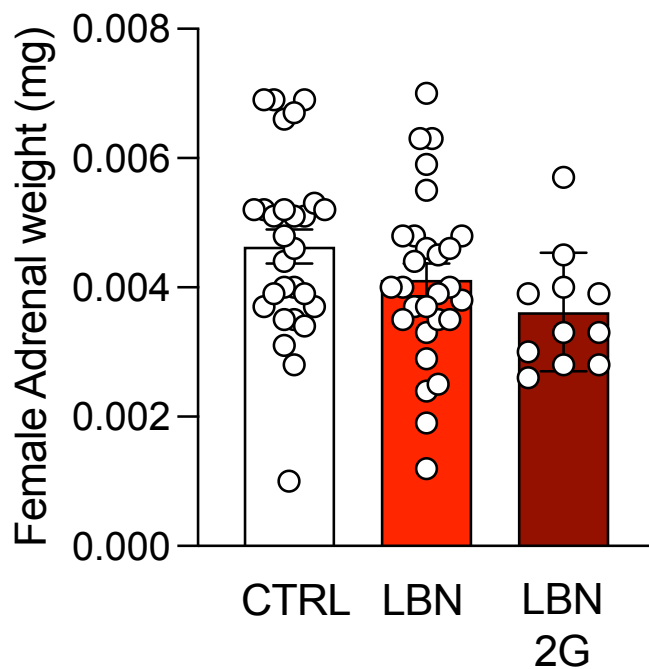

Supplement: Supplement 10 [file media-10.pdf]

A

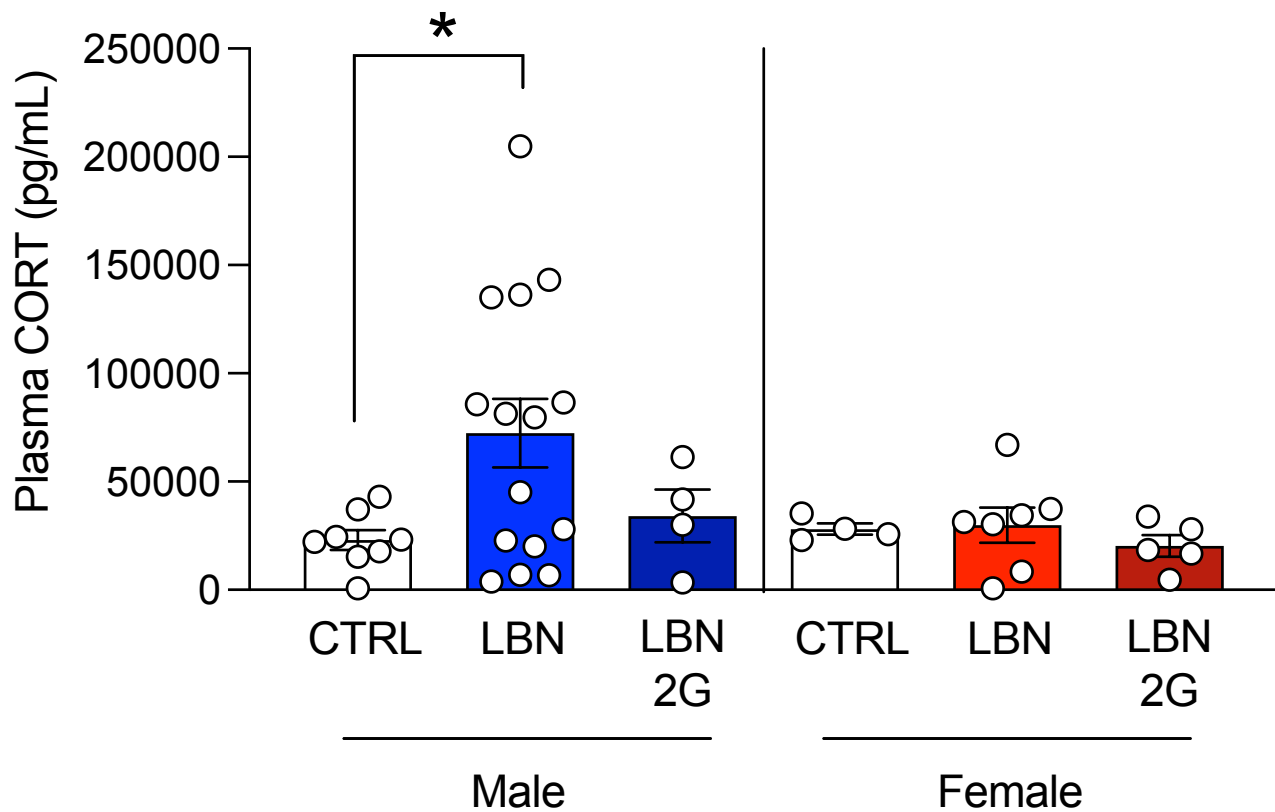

B

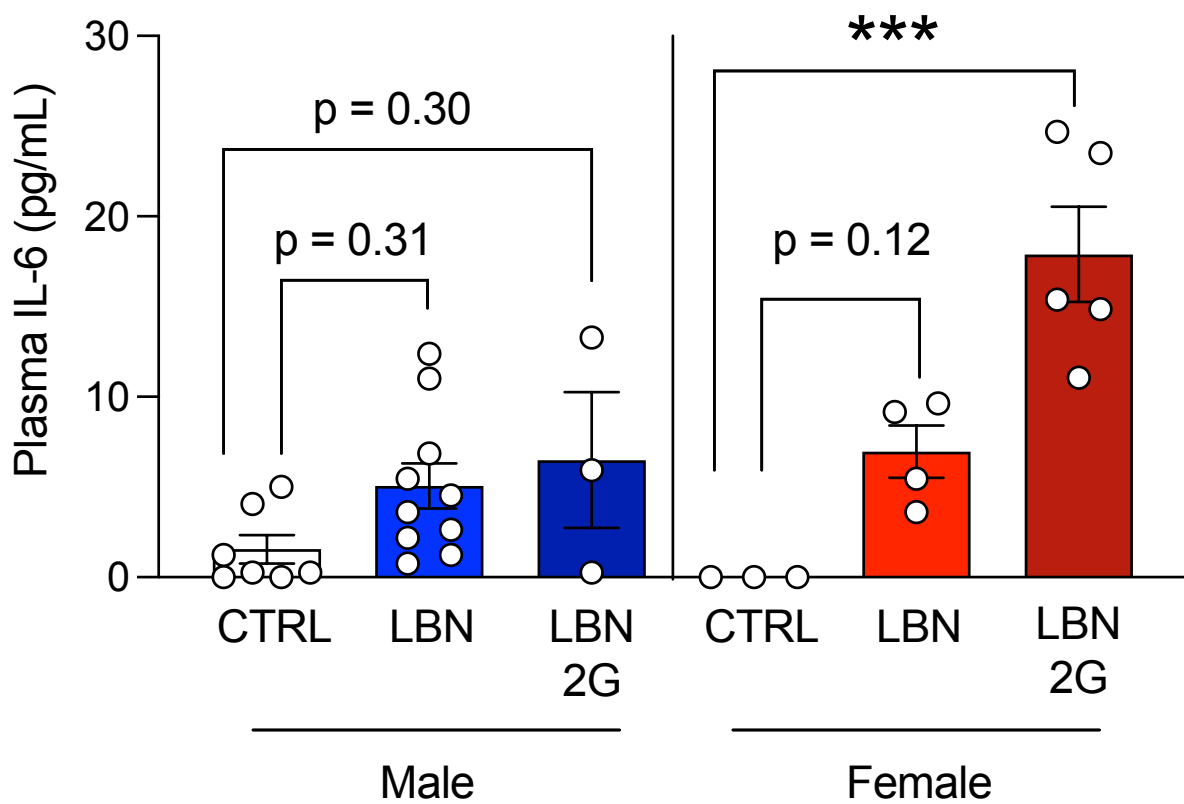

Supplement: Supplement 11 [file media-11.pdf]

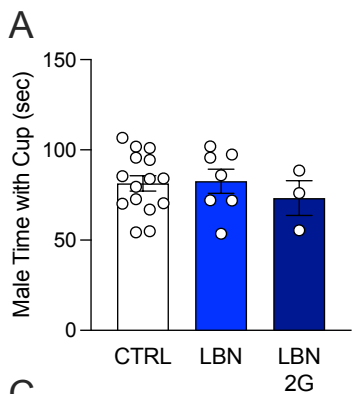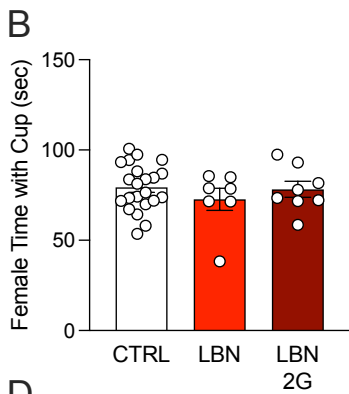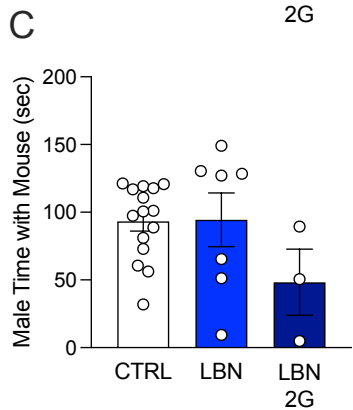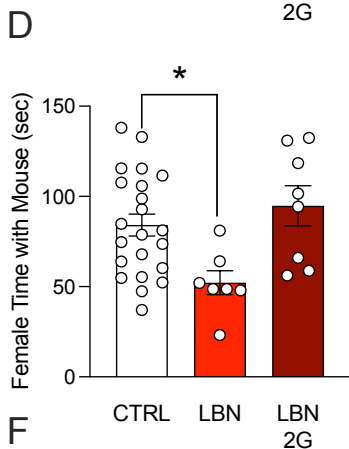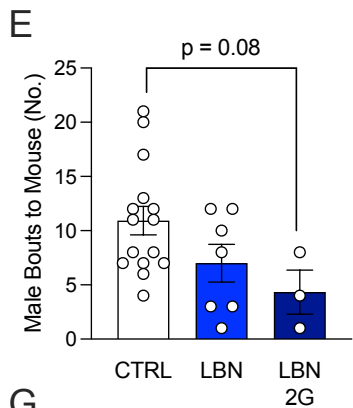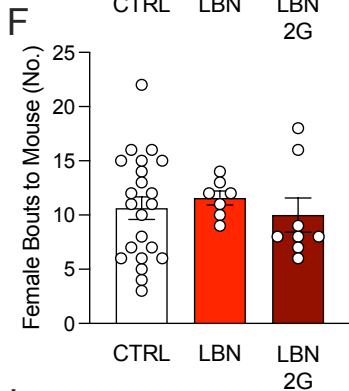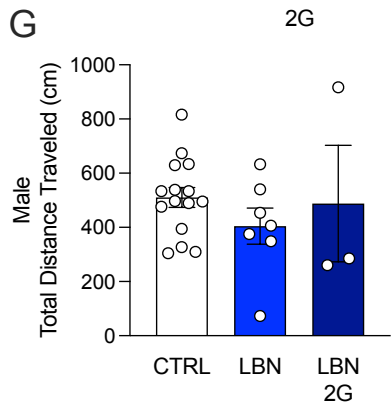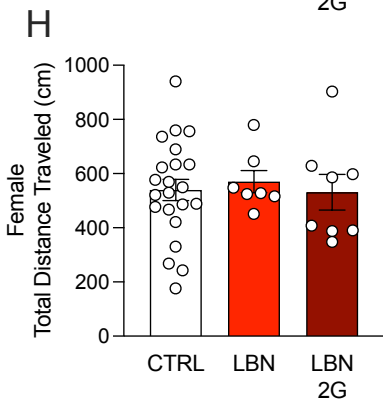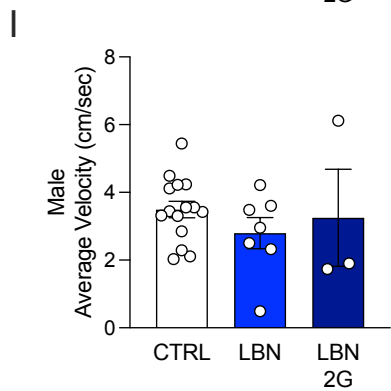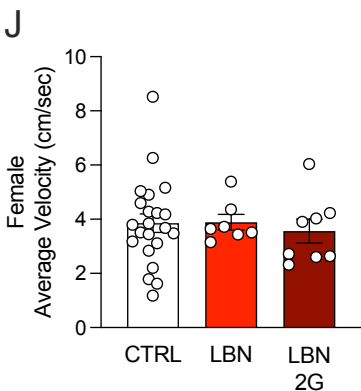

Supplement: Supplement 12 [file media-12.pdf]

**A**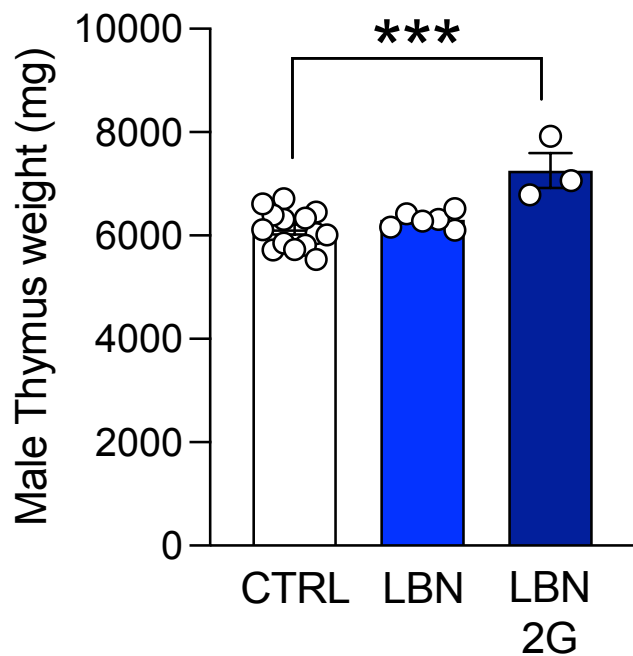**B**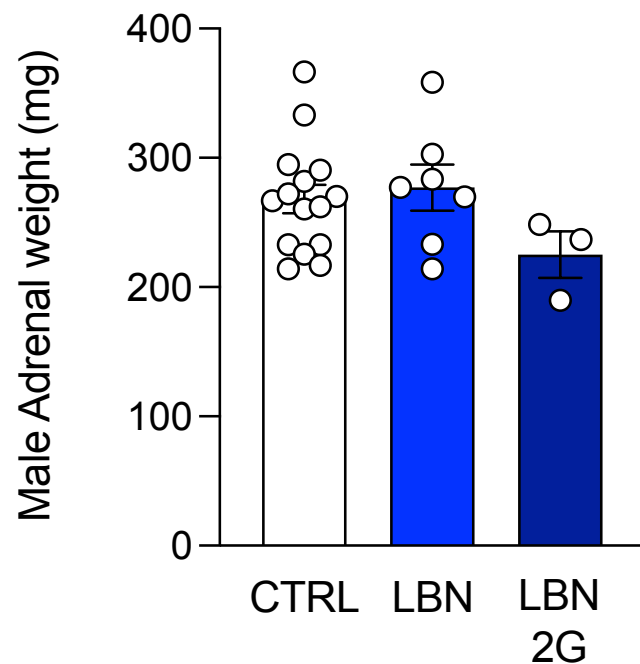**C**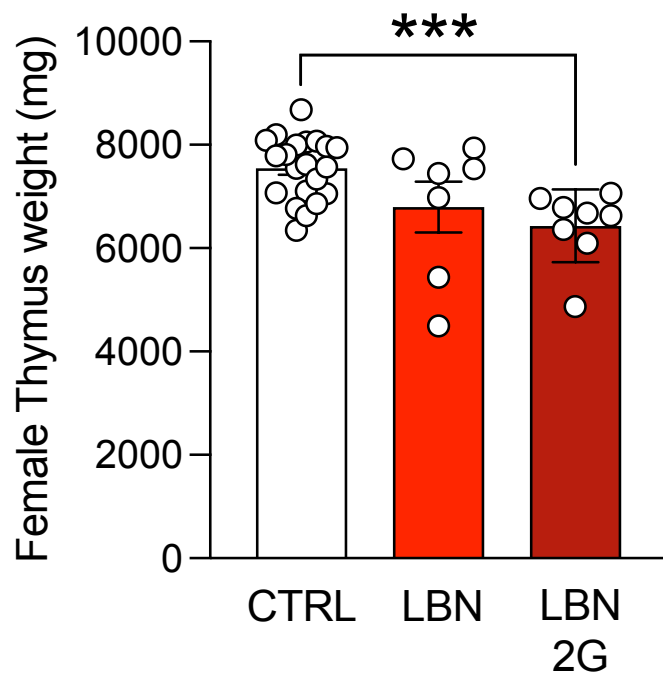**D**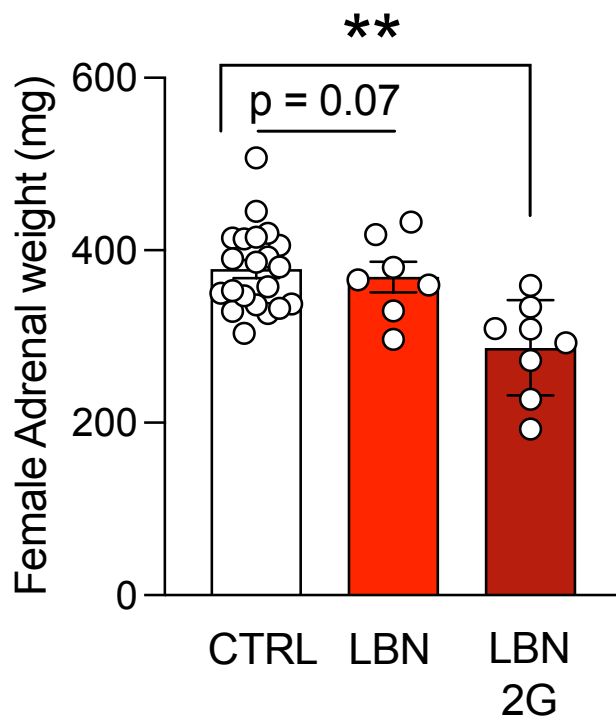

Supplement: Supplement 13 [file media-13.pdf]

Male

Female

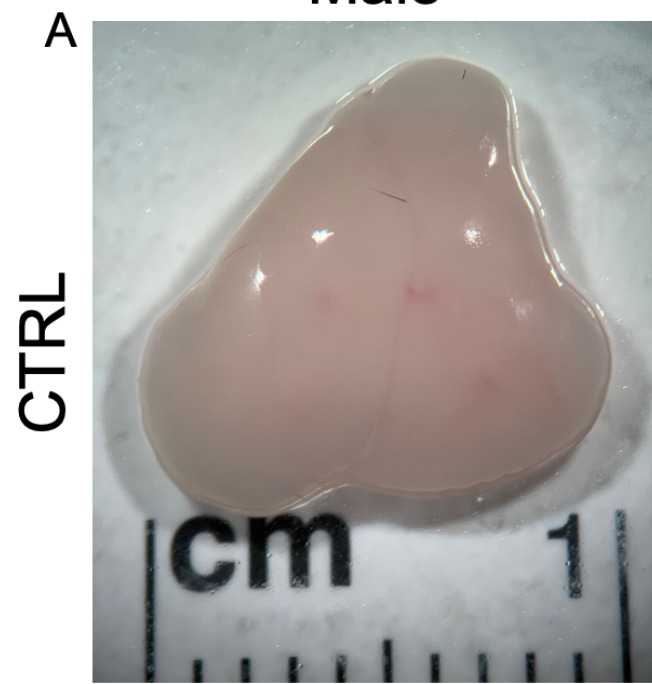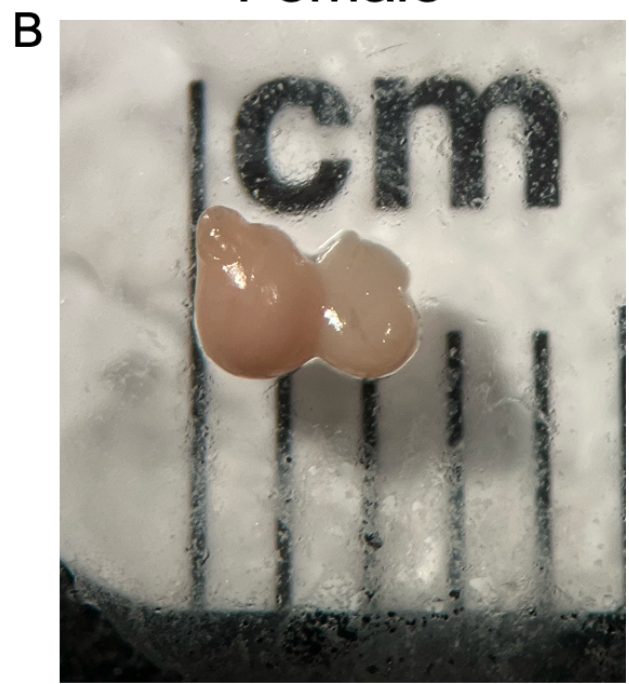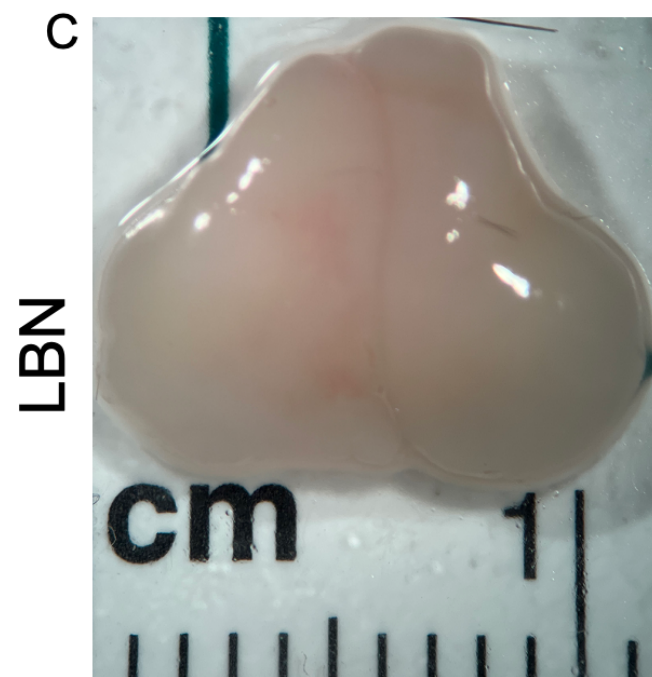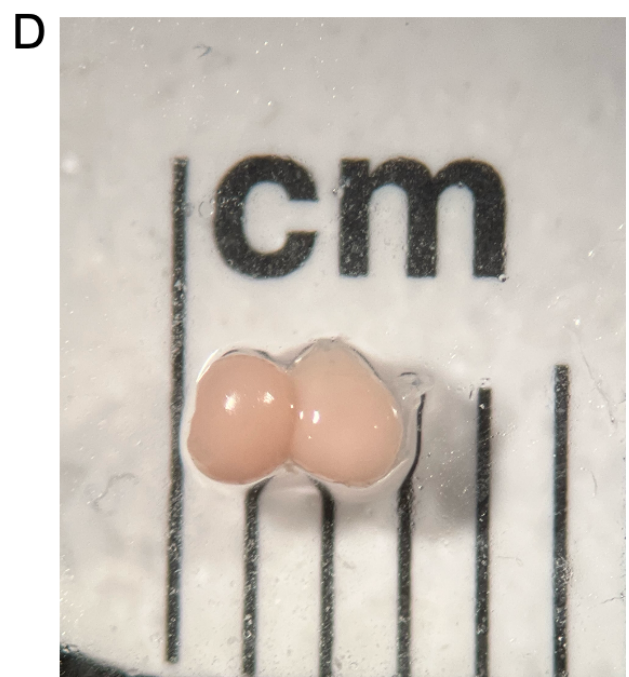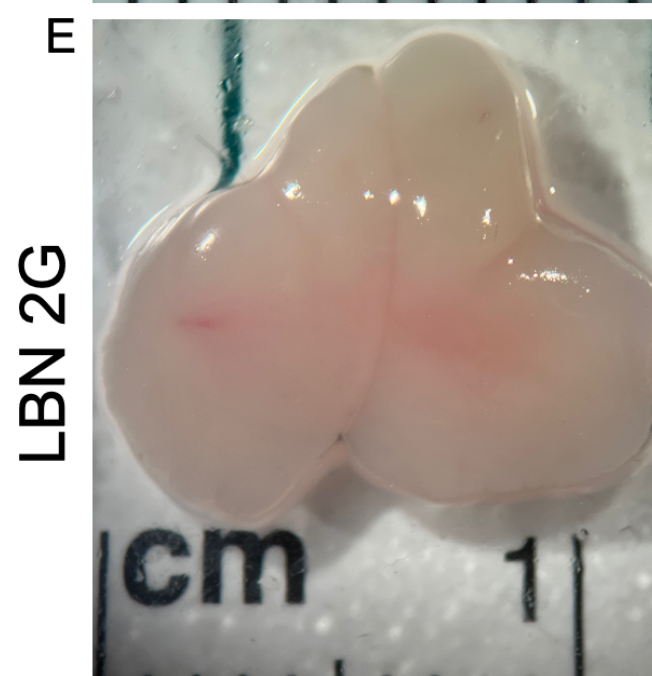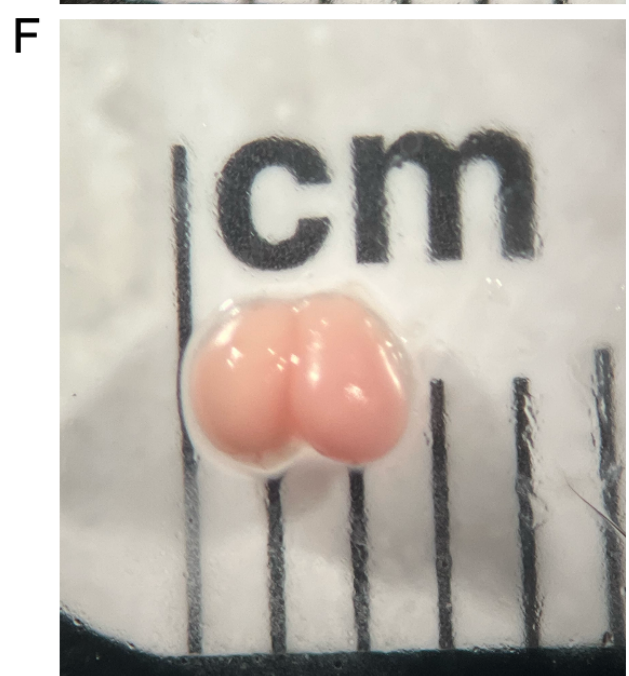

Supplement: Supplement 14 [file media-14.pdf]
