# Supplementary material for: Early life stress induces social behavioral deficits and peripheral biomarker alterations in adolescence that perpetuate intergenerationally
Source: bioRxiv. 2025 Dec 1:2025.11.27.690841. Preprint. [Version 1] doi: 10.1101/2025.11.27.690841 (PMC12694602; doi:10.1101/2025.11.27.690841)
Supplement: Supplement 6 [file media-6.pdf]

A) CTRL Vocalization Spectrogram @ P9

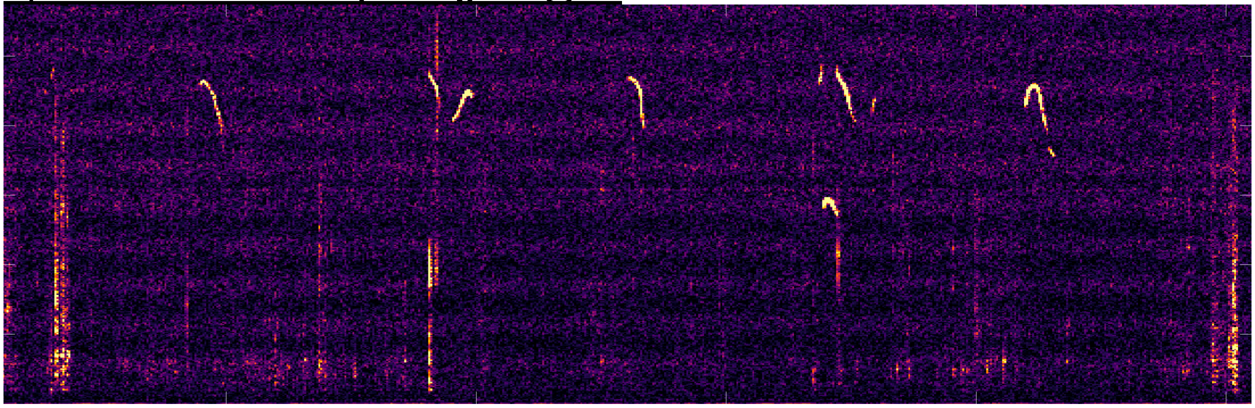

(over 1s time period)

B) LBN Vocalization Spectrogram @ P9

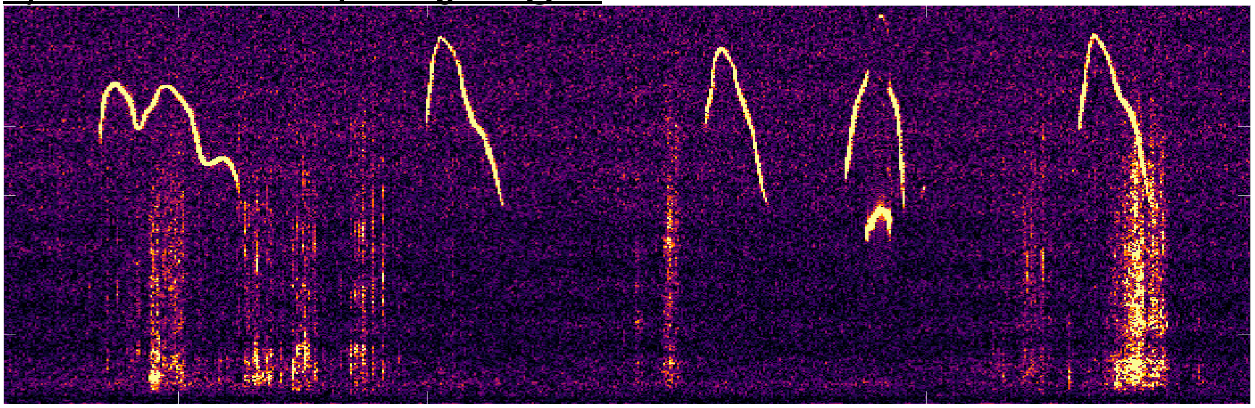

(over 1s time period)

C) LBN 2nd Generation Vocalization Spectrogram @ P9

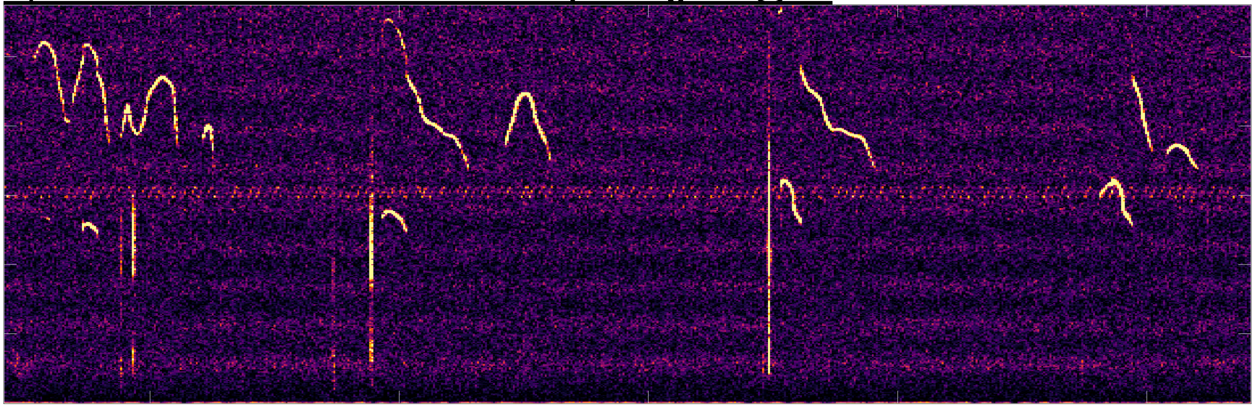

(over 1s time period)
